# The Livelihood of Artisanal and Small-Scale Miners and Awareness of the Use of 3T Minerals in Rwanda—A Case Study in the Rutsiro District: A Qualitative Assessment

**DOI:** 10.3390/ijerph191912570

**Published:** 2022-10-01

**Authors:** Jan Macháček, Martin Schlossarek, Philemon Lindagato

**Affiliations:** 1 Department of Social Geography and Regional Development, Faculty of Science, University of Ostrava, 710 00 Ostrava, Czech Republic; 2 Department of Development and Environmental Studies, Faculty of Science, Palacky University Olomouc, 771 47 Olomouc, Czech Republic; 3 School of Earth Science and Resources, Chang’an University, Xi’an 710054, China

**Keywords:** Rwanda, artisanal and small-scale mining, socioeconomic impact, awareness of 3T minerals, SMART technologies

## Abstract

This article examines the impact of artisanal and small-scale mining (ASM) on livelihood in mining communities in Rwanda (Rutsiro) where wolframite and coltan are mined. The paper discusses the development of ASM and other entrepreneur activities, in particular agriculture. With ASM activities, there is environmental degradation on the one hand but also an improvement in the well-being of the local population on the other. The 3T (tin, tungsten, tantalum) minerals extracted by ASM are used in the electronics industry for products such as smartphones, tablets, and laptops, which are mainly consumed in the developed world. Based on questionnaires and structured research with miners, it was determined how ASM affects their lives, or whether there is a deterioration or improvement in their well-being. The research builds on previous field research in Rwanda. Because of mining, communities in the mining areas have access to health care, they can pay tuition fees, insurance, etc. On the other hand, the lives of miners are endangered by respiratory diseases, accidents in mines, landslides in mining areas, and other negative environmental impacts. The extraction of these minerals, however, may lead to a worse quality of life for the miners responsible for the extraction in developing countries. This different view is also illustrated by the fact that miners themselves often do not know what 3T minerals are used for. ASM benefits miners from an economic perspective but may worsen their quality of life due to unsuitable working conditions. This study covers a broader understanding of socioeconomic impacts of ASM and tries to point out the lack of awareness about the mining of minerals important for the daily use of modern technologies. This article would like to contribute to the larger debate about the lack of awareness of the origin of 3T minerals.

## 1. Introduction

Artisanal and small-scale mining (ASM) is a significant rural non-agricultural activity in the developing world. It is an important source of employment and income for dozens of millions of people and brings economic benefits to further millions who are not directly involved in ASM. According to estimates, at least 134 million people, and according to some estimates, up to 234 million are dependent on this industry [1]. Interest in ASM has increased, particularly in the last four decades, due to growing concern about irreversible landscape interventions, serious environmental disruption, and social aspects associated with mining. The most mined minerals in the African Great Lakes Region (GLR) that are extracted during ASM are 3T minerals, which are processed into tin (cassiterite), tungsten (wolframite) and tantalum. Coltan is an informal trade name for columbite-tantalite ore concentrates that are widely used in the GLR ((Democratic Republic of Congo (DRC), Rwanda, and Burundi) [2]. The extracted minerals then enter the global value chain, and millions of people depend on their extraction, processing, and transportation for their livelihood. These minerals are particularly used in the electronics industry, such as computers, smartphones and tablets in consumer electronics and for smart computing technologies. In addition, these may be used in the automotive, airplane, and medical industries. For example, tungsten is what makes smartphones vibrate, tin is used as a solder on circuit boards, tantalum is used in capacitors, and gold is used to coat wirings [3]. In a globalised world, these products are intended for use in everyday life, to improve the user’s quality of life, and to promote technological development. The use of these components is most visible in the developed world and the world’s largest economies, and in the Smart Economies in Smart Cities [4]. Modern technologies often improve people’s quality of life, and some cannot even imagine their lives without their use. Without 3T minerals, our modern society would not have achieved such technological advancement, and it is questionable whether we would be where we are today without the contribution from 3T minerals. Although 3T minerals promote further technological advancement and improvement in the quality of life in, generally speaking, industrialized countries, the extraction of these minerals in ASM in developing countries is not technologically advanced, and does not necessarily improve the quality of life of those extracting the minerals. ASM often takes place in remote rural areas in predominantly developing countries and is a livelihood for poor and poorly educated people. The absence of modern technology and lack of occupational safety measures are common in ASM. In many cases, ASM involves illegal mining. Due to the above aspects, ASM and extraction of 3T minerals can result in injury to miners, environmental damage, and the deepening of sociopathological phenomena. However, ASM does not only carry negative aspects. In areas where ASM is taking place, ASM-related business activities are developing, increasing the purchasing power of the population, and improving the quality of life in areas near mining sites. So, there are different ways to view this, depending on the perspective used to look at the issue. The greatest irony in the issue of ASM and 3T mining is the (lack of) awareness of the mining processes and the further use of the minerals mined. Most miners who mine 3T minerals have no idea what these minerals are used for and what their further uses are. The same is true of consumers of products that contain 3T minerals. Modern technology users themselves do not know how and where these materials are mined and what is behind the production and processing of the products. This paper aims to bring both perspectives together using the example of the Rutsiro mining site in Rwanda. The research follows upon previous field research that looked mainly at the environmental aspects of ASM [5,6]. The article’s main aim is to focus on the perceptions of the miners on the positive/negative impacts of ASM on their quality of life and their awareness of mined minerals. The authors realize that this question is difficult to answer but this research is being conducted for the first time in the Rutsiro mining site.

### Study Setting and Design 

Importance of 3T Minerals

Approximately 97% of the world’s tin production comes from developing countries and approximately 40% of the world’s cassiterite ore (the main mineral of tin) is mined by artisanal miners [7]. Cassiterite (a tin oxide mineral, SnO_2_) is one of the most abundant tin ores in the GLR, with this region accounting for 3–4% of world production [2]. Due to its mechanical resistance and chemical stability, it accumulates in alluvial deposits from which it is easily separated due to its high weight. Tin is recognised by a number of governments as a critical metal that is strategic to the needs of their technological industries for its use as a tin-indium oxide in electronic touch screens [8]. DRC, Rwanda and Burundi rank amongst the largest producers. Tantalum started to be used in the 1960s in the form of powder, and to make wires for capacitors in the electronics industry. A tantalum capacitor has a high ability to store electrical charge by volume and mass. It is also exceptionally reliable and can operate constantly in extreme conditions for a long time compared to other types of capacitor. These qualities make tantalum capacitors of choice in many critical and industrial applications (e.g., satellites and medical implants), compared to the cheaper but less reliable aluminium and ceramic capacitors. China is among the world’s largest producers of tungsten, accounting for more than 80% of the world’s tungsten ore production [9]. Due to the high demand for products containing tungsten, China, as a producer, is also a consumer of most of its production. Smaller but important mining states include DRC and Rwanda, where production has increased rapidly in recent years, making these mining states among the most important tungsten producers on the African continent. Production in African countries is generally not stable and fluctuates. This is caused by multiple factors, such as world demand, or more precisely the price of raw material, the political situation, and armed conflicts.

The Rwandan 3T mining sector is perceived as an important employer. According to the Labour Force Survey mining employs over 60,000 people [10], whose incomes support more than 170,000 livelihoods [11] or about 2% of the total number of people employed. The majority (about 97%) are, however, informally employed, with only about 1800 formally employed. The relatively low number of people employed in the formal sector is confirmed by the Integrated Business Enterprise Survey (IBES) that estimates that about 5000 people, or 2.3% of the fully formally employed, are within the mining and quarrying sector. In 2018, for example, 21,200 (or 40%) of the 53,300 people informally engaged in mining were also active in subsistence agriculture [12].

## 2. Methods

### 2.1. Study Setting and Design 

**Figure 1:**
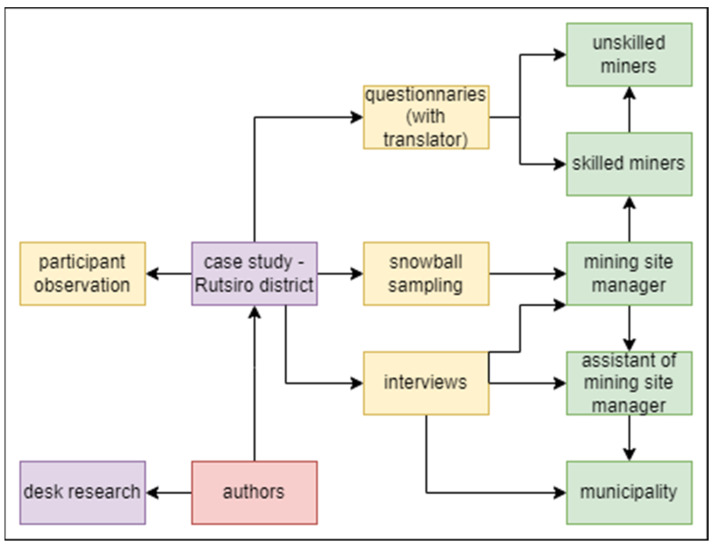
Flow diagram with the steps of the study.

This paper is an associated output of the project “SMART technologies to improve the quality of life in cities and regions” (No. CZ.02.1.01/0.0/0.0/17_049/0008452) [13], which aims to improve urban life by means of modern technologies and the use of SMART methods. People in the developed world today can hardly manage without modern technologies and 3T minerals play an important role in this field. The authors designed multi-stage sampling data collection techniques for this study. Miners and stakeholders were identified, approached, and interviewed through snowball sampling and the support of assistants in the mining company in the Rutsiro mining site. Methodology of snowball sampling is used in similar research by Bansah et al. [14] in Ghana and also by Kouankap et al. [15] in Cameroon, by Arthur-Holmes [16] in Ghana, and by Baffour-Kyei et al. [17] in Ghana and Postma and Geenen in Rwanda [18]. Snowball sampling was chosen due to the suitability of the participants in the case study. It involved the selection of individuals from small, scattered and poorly accessible groups for which selection keys for probabilistic selections do not exist and are difficult to compile and within which contacts occur. The method consists of initially searching for a few people and then contacting those other members of the group who have already been referred by the selected people. The snowball sampling was employed in order to gain access to the miners’ chief, the site manager, miners with long-term skills, unskilled miners, miners who participated in agriculture, and female miners. The task was to create a holistic overview of the researched issue and to capture how the process participants interpreted the situation. Participant observation was another data-collection method used (according to Clifford et al. [19] and Kawulich [20]) in the Rutsiro mining site in Rwanda. The overall research design draws on the theory-confirming case study by Lijphart [21]. The case-selection technique was based on the typical case [22]. For the most recent discussion see George [23]. The interviews were supported by first-hand observations during field visits. Figure 1 shows a flow diagram with the steps of the study.

The topics of the interviews covered five areas: (a) age, family background, mining experience; (b) miners’ perception of mineral extraction and the improvement or deterioration of their well-being; (c) financial remuneration depending on the miners’ experience; (d) perception of environmental and health problems; (e) awareness of the use of mined minerals. The data were fully rewritten and encoded. The authors used Atlas.ti (version 8.4) as text coding and analysis tools. Data were systematically coded, and the transcripts were analysed in an iterative process. Secondary data were collected through extensive document searches, including online sources. Preliminary results were carefully triangulated using various data sources as well as various methods. The Rutsiro mining site represents an example of ASM, and empirical findings from this case study may be generalized for the ASM in Rwanda.

### 2.2. Case Study—Rutsiro District (Rutsiro Mining Site)

The Rutsiro district is in the Western Province of Rwanda, located 150 km northwest of the capital Kigali. The Rutsiro district (1157.3 km^2^) is one of seven administrative units in the Western Province. The district includes 13 administrative sectors divided into 62 areas and 485 municipalities, which accounts for 3.3% of the total number of municipalities in Rwanda. The Rutsiro district has a population of more than 300,000, which is more than 3% of Rwanda’s population. The population density reaches 255 people per one square kilometre [5]. A characteristic feature is a high proportion of the youngest population, where 50% of the population is of pre-productive age, and of which more than 60% of the population is under 25. The main source of income for the population is agricultural activities and mineral extraction. The agriculture industry serves primarily to fulfil a self-sufficiency function, so the locals are thus significantly dependent on the natural conditions that influence these activities [5]. The Rutsiro district is a mountainous area located at an average altitude of 2400 m above sea level. It is characterized by steep slopes and deeply cut valleys, as deep as 200 m. Due to large total rainfall (average annual precipitation reaches 1200 mm), loose slopes are prone to erosion. The season known as “long rains” is from March to April, during which the rainfall is between 40–60% of the total annual precipitation. The long rainy season alternates with the long dry season, which is between June and September. This is followed by a season of short rains from September to December. From December to March there are some shorter dry periods with prevailing days with no rainfall [24]. The Rutsiro mining site was operated by Natural Resources Development Rwanda (NRD) but after privatization in 2015 was split into a few concessions which are under the control of mining companies owned by Rwandans. It is currently undergoing restructuring and mining on a small scale. The total number of miners on the site is between 300 and 400.

### 2.3. Sampling Participants

In addition to interviews and questionnaires, a series of focus groups was organised with artisans, field stakeholders linked with agricultural activities and mining. The interviews were conducted with the help of a translator from the local community, who himself worked in mining, which increased the trust of miners and stakeholders. We collected a total of 43 semi-structured questionnaires and carried out six structured interviews (according to Hay, 2000 [25]) covering key topics from the socio-economic impacts of mining with stakeholders.

The respondents were between 21 and 54 years old, with an average age of 30. 95% of the respondent miners were men. Women can also work as miners, so they do the same hard work as men, including rock digging, however they are under-represented, making up only 16% of total mining workers in Rwanda [26]. The women who worked at the mining site did not participate in actual mining activities but provided ancillary works such as transporting ore for processing and supplying water to the workers at the site. As for the women’s working conditions at the Rutsiro mining site, this is a similar trend to many other countries as noted, for example, by Hinton et al. [27], Bashwira et al. [28], or Arthur-Holmes [16]. More often, however, they work in follow-up processing activities such as panning or separating large chunks of rock from minerals. They and their families are at risk of mine accidents or developing silicosis from exposure to silica dust. In many cases, women have to look after their families and children in addition to working in ASM, thus having dual responsibilities and working harder than men [27]. As argued by Hilson [29], the role of women in the ASM sector varies around the globe, differing both between regions as well as between mines adjacent to each other. In general, women perform worker activities in the ASM sector, such as the actual extraction of rock from the ground, panning, rock washing, and processing of coarse material in processing plants. Another sector where women can work is in the provision of goods and services. In these sectors, women most often work as cooks or salespersons. Less common jobs for women include mine operators or buyers. In many localities, women work in multiple fields. Some may work as cooks while washing rocks by “panning” [30,31]. 

The research took place in October 2020 and April 2021 in the Rutsiro mining site, Rutsiro district in Rwanda. All ethical research-related issues were addressed prior to data collection [32]. The research participants signed informed-consent forms prior to the start of interviews. In cases where study participants did not want to sign written informed-consent forms, they were given an option to confirm their involvement by verbal consent, which was recorded officially by the researchers. The involvement in the study was voluntary with participants being able to leave or interrupt the interview at any time. Consent was sought from participants before interviews and field notes were recorded. The research results were anonymised, so that more detailed information about individual participants could not be obtained.

## 3. Results

### 3.1. Main Findings

Almost 90% of the interviewed miners reside within an hour’s travel time from the mining site and are mainly from the nearby communities. In terms of marital status, 30% of the miners are single and 70% are married. The average size of a miner’s family is four members, with half of the families having two family members working in mining. Most of the miners worked in agriculture before and some of the miners started working in mining right after finishing primary school. Some miners worked in construction before mining but switched to work in the mining sector due to higher wages. The education level of miners was low, reflecting the education level of the population in rural areas. Over 73% of the miners have only primary school education; the remaining 27% have secondary school education.

The average number of years a miner has worked in mining is seven years. Over 80% of the miners interviewed work in mining year-round, and in the “traditional ASM” type. According to Wall [33], this is a type of mining where the minerals have long been known to occur, and mining activities have been on-going for several generations. Income from mineral extraction is a key part of the livelihood of the locals. Mining rights (if any) and mining skills are passed down from generation to generation, and most family members participate in mining activities. Only 20% of the miners work occasionally, thus falling into the “seasonal ASM” type of mining. According to Weber-Fahr et al. [34], it is more of a secondary source of livelihood, supplementing the source of income generated by agricultural activities. Mineral extraction takes place during periods that are not favourable for agriculture. In some areas, individual family members or the whole family temporarily migrate to areas where minerals are found. Once the agricultural season begins, the people move back to the fields. It is also common for individual family members to divide and work in both the mining and agricultural sectors.

### 3.2. Social-Economic Aspects of ASM in Rutsiro Mining Site

**Figure 2:**
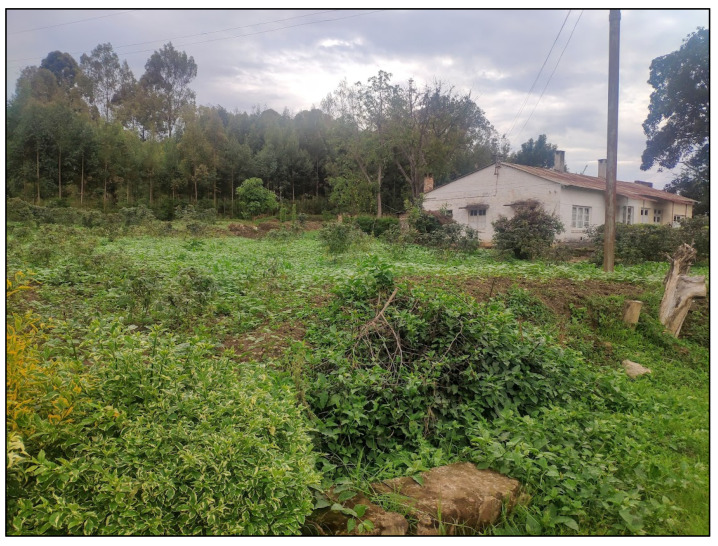
Unused land converted to agricultural land due to the COVID-19 pandemic. Photo taken by Jan Macháček, one of the authors.

Mining is the main source of income for 80% of miners. The average monthly salary of a miner is Rwandan francs (RWF) 45,000 (RWF 1000 ≈ USD 1 as of November 2021), with the lowest salary RWF 30,000 per month and the highest salary RWF 70,000. Miners are paid an average of RWF 2500 per kilogram of cassiterite. The RWF miner salary commonly increases with age or experience. With higher income, miners can afford paying health insurance for themselves and their families, school fees for their children, and they can also provide for basic necessities. Most of these miners depend on mining as their only source of income. A fifth of miners combine mining with agriculture. Crops grown include corn, cassava, carrot, tomatoes, and beans. With the money earned, a miner can buy livestock such as goats, sheep, and chickens. With higher salary, a miner can then buy a cow, which is historically a symbol of wealth and status in this region. The cow is also a source of livelihood, additional potential profit, and income diversification. The money from mining will therefore help the miner to diversify his income and earn more. The more prosperous workers can then buy more cows or fields to diversify their farming activities, and/or build a house with a better quality (tin) roof. All this contributes to the economic development of the area and to the rising standard of living of the locals. An interesting finding, which was particularly influenced by COVID-19, is the expansion of the area used to grow crops using the concession. The first site visit was organized in October 2020. Many areas within the mining site contained native and newly planted forest. In addition to these forested areas, there were areas with seeding of other tree species as well as transitional areas between uncultivated land and forest. During the second visit in April 2021, much of this unused land had been converted to agricultural land, mainly due to COVID-19 restrictions. According to the mining site manager, as much as 20% of the unused land had been converted to agricultural land due to potential restrictions and thus limited food supplies (Figure 2), increasing food subsistence and thus ensuring greater food self-sufficiency for miners residing at the mining site. Interviews with other stakeholders revealed that ASM can increase the purchasing power of the population, increase demand for local products, contribute to increasing foreign exchange earnings, reduce population migration to urban areas, and enable the exploitation of mineral deposits by other stakeholders. Most miners perceive ASM as a positive contribution to their lives, especially in terms of higher income.

### 3.3. Health and Safety Aspects of ASM in Rutsiro Mining Site

When asked if they perceived any negative impacts of ASM on their well-being, 85% of the respondents mentioned health problems caused by moving around in shafts and working in a dusty environment as a big problem. 65% miners used the term ‘silicosis’ and others reported that they had lung problems. Silicosis is a form of occupational lung disease caused by inhalation of crystalline silica dust. However, apart from silicosis, miners can also develop various types of bronchitis. Other miners mentioned that, apart from silicosis, they risked injury, or in the worst case, death caused by cave-ins. According to The New Times, Rwandan Daily Journal in 2019 at least 50 miners died, and 41 sustained injures in this area [35]. However, they did not rate the risk of landslides or cave-ins as significantly as silicosis. Some miners complained of back problems and headaches. The health and safety issues that put miners at risk are listed in Table 1. In bold text are hazards that were identified by miners in Rutsiro; according to our research, there are similarities between health and safety hazards mentioned by Drechsler et al. [36].

When the miners were asked about the negative aspects of ASM, most respondents pointed only to health complications. None of the miners indicated that ASM had a negative impact on, for example, the environment. However, in some mining communities, these impacts are not associated with mining itself but are rather seen as an intensification of natural processes [5,6].

However, the downsides of ASM mainly include the environmental aspects associated with mining which are of a longer-term nature. None of the miners mentioned these aspects. The negative environmental impacts of ASM, on the other hand, are mentioned by stakeholders other than miners [5,6]. Mining activities are thus connected with illegal deforestation, water pollution, and soil contamination. Deforestation leads to landslides and accelerated erosion [5,37]. However, the environmental aspects of mining were not a focus of this research, but may be the subject of a future study.

### 3.4. Awareness of the Use and Extraction of 3T Minerals

The most interesting finding, however, was a lack of awareness of miners when it came to the use of the minerals they extracted. As noted in the introduction, 3T minerals have wide application in many industries and play an irreplaceable role in technology-intensive industries. Some of the interviews with miners were directed towards the exploitation and further use of minerals extracted by them. Due to the presence of an interpreter, we could accurately record the miners’ responses, from which we can see that almost no miner knew the majority use of 3T minerals. Half of the miners did not know what the minerals they were extracting were used for. Some miners said that although they did not know what the minerals were used for, they knew they were transported to Kigali and from there abroad where there was a market for them. Several mentioned Mombasa, some even Malaysia (there are tin smelters in Malaysia). The other half of the miners then reported that the minerals are used to make steel, railway sleepers, shipping containers, and for making construction tools such as shovels, pickaxes, and hoes. Several miners reported that minerals are used to make cutlery. Thus, none of the miners listed either of the primary uses, in this case, of wolframite and coltan. Just as the miners do not know what the minerals they extract are used for, consumers know little or nothing of the origin and extraction of minerals. Efforts to create awareness and increase knowledge of the origin of 3T minerals have been ongoing for several years. However, these are mainly efforts to prevent the use of conflict-minerals by global companies (see Dodd-Frank Wall Street Reform and Consumer Protection Act of 2010 [38] or Conflict Minerals Regulation [39] by the EU). Fortunately, not all minerals mined by ASM fall into the conflict minerals category. However, there is very little awareness of the issue of conflict-free minerals and ASM amongst consumers. 

## 4. Discussion

ASM is the most important non-agricultural rural livelihood in sub-Saharan Africa [40]. The sector provides employment to the otherwise unemployed [41] and provides income in areas where agriculture is unviable [42,43]. In these areas, mining represents one of the main sources of income for the local population, thus forming an important pillar of economic development, since mining activities are related to other employment opportunities such as further mineral processing, tagging, transport, as well as follow-up services for workers in the surrounding mining site. Most miners work only in mining, and a smaller proportion combine mining mainly with agricultural activities. The more prosperous miners, especially the older ones who have worked in mining for a long time, can afford to acquire livestock and fertilizer for more efficient land cultivation. They can resell the acquired and surplus livestock and crop products. The most common products include meat, milk, eggs and beans, corn, and cassava. As Cook et al. [44] note, an example of this is a miner who was able to acquire three cows due to his mining salary. A dairy cow produces up to five litres of milk per day, the purchase price of 1 litre of milk is RWF 200, which amounts to RWF 90,000 per month, whereas the monthly costs associated with these three cows are RWF 35,000 per month, resulting in a monthly income of RWF 55,000. The same study [44] documents that in mineral-rich areas, mining is the dominant source of income for most workers and has become increasingly important over the past decade. Most miners claim that mining offers an above-standard source of income. These findings are similar to those of the Perks study [45]. It is possible to earn up to twice as much money in mining areas than in agriculture. A miner’s salary is half that of construction workers in the capital of Kigali [44]. ASM has a domino effect on local economies because most of the funds are reinvested in the region of interest. Despite the small scale of mining activities, its importance to the social and economic development of the region is much greater. ASM generates important local purchasing power and leads to demand for goods and services. The locals, who are completely or partially dependent on ASM, need to diversify their income. Diversification is a way to make income earned from ASM sustainable, and have money [41,46] in case of emergencies such as adverse drought, or e.g., COVID-19. The threat constituted by mineral deposit depletion or declining reserves can also be a major problem for sustainable ASM [47]. Even illegal mining has a positive impact on the local economy and small business activity. Income from this illegal activity goes back to local communities, thus increasing community capital. The problem with illegal mining is that some miners do not have any identity documents or work permits from the concession holder in the area. These miners have no health insurance or access to health care. They often do not even have work boots and helmets and basic occupational safety devices available. Apart from the aspects associated with illegal mining, ASM also brings along other negative impacts, among which the miners themselves list health problems such as various types of bronchitis and silicosis as well as injuries caused by the collapse of a part of the shaft or cave-ins. The above health and safety problems of miners correlate with the research by Elgstrand et al. [48], who among other things, report that some mines where light mechanisation is used are equipped with stone-crushing lines. The constant noise and high dust levels lead to health complications caused by poor and inadequate sleep. As argued by Drechsler et al. [36], ASM puts miners at much higher health and safety risks than they realize. Hentschel et al. [49] and Smith et al. [50] make similar points. In relation to these negative aspects, social pathologies such as work under the influence of drugs or alcohol, and increased crime occur at mining sites [14,51,52]; moreover, illegal mining is fraught with violence and unrest between competing and rival groups of illegal miners.

The negative impacts of ASM on local populations, miners, and dependants on mining could be mitigated by greater global public awareness of 3T minerals. The paradox of the ASM industry is that most people who depend on the industry do not know where these minerals end up and what they are used for. The same is true for modern technology users from the developed world, who use products containing 3T minerals daily. Most of these consumers have no idea that, by using products containing the minerals, they themselves are contributing to the environmental destruction and, in many cases, the health of the population dependent on mining. The consumer awareness of the beneficiation of 3T minerals has been raised and enhanced since the emergence of the certification for conflict-free sourcing of minerals. Tantalum-Niobium International Study Center (TIC) identified a target to increase awareness and promote the remarkable properties of tantalum and niobium, and disseminate relevant information to the stakeholders among its members [53,54]. This effort of raising awareness of the 3T minerals among suppliers has been well adopted and is continuously enhanced [55]. The awareness of the Rwandan mining sector was raised in line with the Extractive Industries Transparency Initiative (EITI) mission, but has been evident only in articles and studies [56]. Unfortunately, neither miners nor users like to read. Thus, they both remain unaware of the benefits of using 3T minerals. This outcome seems to be not surprisingly, because some of the mining workers in Rwandan start working without having prior knowledge of minerals [26]. The worldwide initiative of The International Tin Supply Chain Institute (ITSCI) Programme for Responsible Mineral Supply Chains operates in Rwanda in all mines. But according to the authors’ findings in the Rutsiro district and another report [57], there are several indications that ITSCI tags are used to tag production that has not been sourced in ITSCI mines and hence the ITSCI Programme has shortcomings and does not function well in many cases. There is evidence that in the Rutsiro mining site, informal miners who are not members of the mining cooperatives steal the local mineral production. According to the Rwanda National Police, the Rutsiro mining site operates with illegal mineral traders who buy minerals from thieves and illegal miners [58]. Having less knowledge of the applications of 3T minerals has positive impacts only on consumers and suppliers, because the price remains relatively low due to the cheap labour that is traditionally associated with ASM. Once the employees/small mining workers know how important 3T minerals are, the cost of extracting the minerals is likely to escalate. They may claim the high wages which, in return, can increase the price of the final products (notably electronic devices). If the awareness of 3T minerals is focused and becomes public knowledge, there is a risk of creating instability in mineral supply chains which can be covered by shifting production patterns to other technology areas that attract relatively low public attention [59]. According to the historical evidence, users are unlikely to have awareness of neither the mineral content nor the origin of the products that they are using [60]. If they did know, it would not change much either in supply chain or in mining activities, because they appear not to care about the effects of consumption [61]. One of the few studies addressing this issue is the article by Boluda et al., 2021: What do Computer Scientists Know About Conflict Minerals? [3]. As stated by these researchers [3], no research so far has been carried out to investigate people’s and users’ awareness of this topic. For this reason, Boluda et al., conducted research to explore the level of awareness of 3T minerals (their extraction, processing, and finished product) and their social and environmental impacts. Their research sample was 135 participants. Most of the participants were between 46 and 55 years old and most were university educated with either a PhD, Master’s, or Bachelor’s degree. In addition, most were from technology, computers, or engineering sectors. It is evident that the researchers selected a group of specialists who are close to these technologies professionally and who were assumed to be more informed about 3T minerals than the general population. The research results showed that most computer professionals were not familiar with these issues. It was also found that participants knew more about the use of minerals in electronics than they did about their socio-environmental impacts. As for individual minerals, participants knew more about gold than the other three minerals [3]. In general, it can be concluded that overall awareness of people employed in the sectors that are relatively close to 3T minerals is very low. This poor awareness of the beneficiation of 3T minerals is evident across worker populations at very different levels, e.g., the technology professionals who participated in the Boluda et al. study [3], as well as the ASM miners interviewed for our Rutsiro mine case study.

The above activities, political decisions, and new legislation on the part of supply chains may help to improve the conditions of miners and dependants on mining in the years to come. A big step must also be taken by local governments to enforce environmental laws, safety practices and to work more with the local community.

## 5. Conclusions

In this paper, the authors attempted to analyse the positive and negative aspects that ASM brings to the local populations using the example of the Rutsiro mining site in Rwanda. A significant and relatively simple step to help improve health and safety standards is monitoring and controlling mining areas to ensure that mining companies comply with all standards, regulations, and laws. Rwandan mining and environmental laws are (in comparison with other GLR countries) of a high standard and consider the economic benefits of mining and the preservation of environmental standards. More education of the end-product buyers could help mitigate the negative aspects. The irony of ASM is that the miners who extract the minerals do not know what the material is used for. The same can be said about users and the global public, who are largely unaware of the impact that the use of products containing 3T minerals has on people in the world’s poorest countries. The paper aims to emphasize the issue of ASM in terms of social impacts, and to contribute to a discussion that would result in greater awareness among users of modern technology, i.e., all of us for whom modern technology enhances the quality of life. The authors intend to follow this investigation with research that looks in more detail at the awareness of ordinary modern technology users about the mining and processing of 3T minerals.

## Figures and Tables

**Table 1:** Summary of health and safety risks related with ASM.

Common Health and Safety Hazards	
Types of Health and Safety Hazards	Examples of Potential Hazards
1. Chemical hazards Battery acid and solvents Diesel fuel, petrol, oil Chemicals used in mineral processing Exhaust fumes	**Respiratory problems** Skin rashes and diseases Poisoning and death Chemical skin burns **Loss of eyesight** **Suffocation and death**
2. Biological hazards **Bacteria, viruses, pathogens** **Dust and moulds** **Parasites and insects** Poisonous snakes and spiders Wild animals	Diarrhoeal diseases such as cholera and typhoid fever **Allergies, asthma, or lung diseases such as silicosis** Skin rashes Poisoning Infected wounds
3. Physical hazards Heat and sunlight **Vibration** **Noise** Radiation Flooding **Landslides** Flying rock fragments Dangerous use of explosives	Body overheating or heat stroke **Hand-arm vibration syndrome** **Hearing loss** Drowning and death Broken necks, arms, legs Cut wounds Death
4. Hazardous working conditions Repetitive motion Heavy loads **Uncomfortable body** positions Machines with moving parts Electric current	Chronic back pain Muscle stiffness Numbness in fingers and hands Loss of finger or hand Electric current (e.g., from damaged equipment) Loss of eyesight
5. Stress-related hazards Sexual, physical, or verbal harassment Exploitation **Work in shifts** Alcohol or drugs **Long working hours**	Depression, low self-esteem, exhaustion Malnutrition Stress-related illnesses Injuries resulting from violence Rape Power outage and fall-related injuries

Drechsler et al., [36]., adapted by authors.
